# Reduced frequencies of Foxp3^+^GARP^+^ regulatory T cells in COPD patients are associated with multi-organ loss of tissue phenotype

**DOI:** 10.1186/s12931-022-02099-2

**Published:** 2022-07-02

**Authors:** Jia Hou, Xia Wang, Chunxia Su, Weirong Ma, Xiwei Zheng, Xiahui Ge, Xiangguo Duan

**Affiliations:** 1 Department of Respiratory and Critical Care Medicine, General Hospital of Ningxia Medical University, Ningxia, China; 2 Ningxia Medical University, Ningxia, China; 3 Department of Pathogen Biology and Immunology, School of Basic Medical Science, Ningxia Medical University, Ningxia, China; 4 Department of Respiratory Medicine, Seventh People’s Hospital of Shanghai University of TCM, Shanghai, China; 5 College of Clinical Medicine, Ningxia Medical University, Ningxia, China

**Keywords:** COPD, T-regulatory cells, GARP, Emphysema, MOLT

## Abstract

**Background:**

Expression of glycoprotein A dominant repeat (GARP) has been reported to occur only in activated human naturally occurring regulatory T cells (Tregs) and their clones, and not in activated effector T cells, indicating that GARP is a marker for bona fide Tregs. A different phenotype of chronic obstructive pulmonary disease (COPD) may have a different immunologic mechanism.

**Objective:**

To investigate whether the distribution of Tregs defined by GARP is related to the multi-organ loss of tissue phenotype in COPD.

**Methods:**

GARP expression on T cells from peripheral blood and bronchoalveolar lavage (BAL) collected from patients with COPD was examined by flow cytometry. The correlation of GARP expression to clinical outcomes and clinical phenotype, including the body mass index, lung function and quantitative computed tomography (CT) scoring of emphysema, was analyzed.

**Results:**

Patients with more baseline emphysema had lower forced expiratory volume, body mass index (BMI), worse functional capacity, and more osteoporosis, thus, resembling the multiple organ loss of tissue (MOLT) phenotype. Peripheral Foxp3^+^GARP^+^ Tregs are reduced in COPD patients, and this reduction reversely correlates with quartiles of CT emphysema severity in COPD. Meanwhile, the frequencies of Foxp3^+^GARP^−^ Tregs, which are characteristic of pro-inflammatory cytokine production, are significantly increased in COPD patients, and correlated with increasing quartiles of CT emphysema severity in COPD. Tregs in BAL show a similar pattern of variation in peripheral blood.

**Conclusion:**

Decreased GARP expression reflects more advanced disease in MOLT phenotype of COPD. Our results have potential implications for better understanding of the immunological nature of COPD and the pathogenic events leading to lung damage.

**Supplementary Information:**

The online version contains supplementary material available at 10.1186/s12931-022-02099-2.

## Introduction

Chronic obstructive pulmonary disease (COPD) is an inflammatory lung disease related to an abnormal immune response that results in airflow limitation. Although cigarette smoke exposure is the most common risk factor for COPD, pulmonary and systemic inflammatory responses persist after smoking cessation in COPD patients [1], suggesting non-environmental factors are driving inflammation in susceptible individuals. Cumulating evidence show that cigarette smoking can generate new self-antigens that may trigger the activation of abnormal "autoreactive" immune responses [2, 3]. Hence, the immunoregulatory function may determine the susceptibility to, and severity of, COPD.

Naturally occurring CD4^+^CD25^+^ regulatory T cells (nTregs) are a distinct subpopulation of lymphocytes that play a critical role in controlling immune responses and promoting and maintaining self-tolerance. nTregs must depend on the forked-head helix transcription factor (Foxp3) for differentiation and development as well as maintenance of function, and the identification of Foxp3 facilitated murine nTreg identification [4]. Foxp3 has been regarded as a specific molecular marker for Treg cells. However, human effector T cells have their Foxp3 expression significantly upregulated upon activation, and such cells do not have immunomodulatory capacity [5]. Therefore, the use of Foxp3 as a specific marker for human nTregs is increasingly questioned [6]. Some other common surface markers used to identify nTregs such as CD25, cytotoxic T-lymphocyte antigen 4 (CTLA-4), and glucocorticoid-induced tumor necrosis factor receptor (GITR) encountered the same problem, i.e., these markers are constitutively expressed, not only in nTregs, but also in activated effector T cells [7–9]. Likewise, both nTregs and activated effector T cells show decreased expression of CD127. The search for surface markers specific for human nTregs has been an important topic in this field [5, 10, 11]. It is uncertain whether molecular markers that are specifically expressed in nTreg cells exist.

The breakthrough in this field has been the discovery of the glycoprotein A dominant repeat (GARP or LRRC32). Using DNA microarray technology, Unutmaz et al. [12] discovered for the first time that activated human Treg cells express a transmembrane protein, GARP, which is not expressed by activated or resting effector T cells. GARP is not induced to be expressed on activated effector T cells, which have high levels of Foxp3 expression induced by TGF-β stimulation but lack suppressive function. GARP mRNA is barely detectable in freshly isolated CD4^+^ T cells, but GARP mRNA levels rise rapidly in Treg following T cell receptor (TCR)-mediated activation, whereas they do not occur in non-Treg cells [13–15]. In addition, high GARP expression distinguishes Treg cells from interleukin 17 (IL-17)-secreting cells [12]. Forced expression of GARP in non-Treg cells using transgenic techniques results in the acquisition of some or all the phenotype and function of Tregs (including Foxp3 expression). Knockdown of GARP in nTreg cells results in a significant decrease in suppressive activity, while Foxp3 expression does not change. Thus, GARP is a highly specific and functionally relevant marker molecule expressed by activated nTreg cells and has been regarded as a potential target for immunotherapy [16–19].

In this study, we aimed at investigating whether there was abnormal distribution of bona fide activated Foxp3^+^ regulatory T cells identified by GARP in the blood and BAL of patients with COPD, and whether this distribution was associated with clinical phenotype of COPD. The findings should provide new insights into the underlying mechanisms of persistent inflammation and adaptive immunology in COPD.

## Materials and methods

### Patient enrolment and study design

Forty-three patients with COPD were recruited at the General Hospital of Ningxia Medical University, China. Fourteen never-smokers and 12 smokers (> 20 pack-year history) with normal lung function were also recruited as controls. COPD diagnosis and severity classification were performed according to the criteria of the Global Initiative for Chronic Obstructive Lung Disease (GOLD) [20]. Patients having a history of asthma and/or allergic rhinitis, or suffering from infections, malignancy, autoimmune disorders, and other immune-related diseases were excluded. Patients on antibiotics, systemic steroids, chemotherapeutics and immunosuppressors within the four weeks before the enrollment, were also excluded. None of the COPD patients had experienced respiratory infection or acute exacerbation over the last four weeks prior to the study.

The study was approved by the Ethics Committee and Medical Faculty of Ningxia Medical University (approval no. 2020–678) and conducted in accordance with the Declaration of Helsinki regarding human medical research. All the subjects provided written informed consent prior to study participation.

### Computed tomography (CT) scan quantification of emphysema

All COPD patients underwent at least one high-resolution chest CT scan using multidetector-row scanners (Siemens Healthcare GmbH, Germany). Detailed CT protocols have been described elsewhere [21]. Quantitative analysis of emphysema is based on the proportion of CT voxels below − 950 Hounsfield units (low attenuation area, LAA) using the software of 3DSlicer (http://www.slicer.org).

### Cell collection and processing

Peripheral blood samples from each subject were collected in EDTA treated tubes and were processed to isolate peripheral blood mononuclear cells (PBMCs) for flow cytometry procedures. Blood samples were layered onto Ficoll-Paque Plus (Amersham Biosciences), then centrifuged (400×*g* for 20 min at 22 °C), and PBMCs were harvested. PBMCs were washed twice in PBS solution at 300×*g* for 5 min at 4 °C, then were resuspended and viable counts obtained.

Sequential BAL preparation was performed and processed as previously described  [22]. To avoid the acute effects of smoking, smokers were asked to stop smoking at least 12 h prior to the BAL procedure. Using a wedged flexible bronchoscope (Olympus BF-290, Tokyo, Japan) under topical lidocaine, four separate 50 mL aliquots of sterile 0.9% saline were infused into one segment of the right middle lobe and gently suctioned. Since the lavage fluid from the first 50 mL aliquot is known to contain cells and material from the central airways rather than the peripheral bronchial and alveolar, the lavage fluid from the first aliquot was discarded. The leftover lavage fluid was filtered and resuspended at 10^6^ cells/ml in RPMI-1640 medium at 4 °C after being washed twice in phosphate-buffered saline. All BAL and peripheral blood samples were processed immediately after sampling.

### Flow Cytometry Analysis Software (FACS) analysis

Freshly isolated PBMCs were stained and incubated with relevant antibodies for 30 min at 4 ℃ in a dark room. Cells were washed twice before being analyzed by a BD LSRII flow cytometer (BD Biosciences, San Jose, CA, USA). For intracellular cytokine staining, cells were activated by phorbol myristate acetate (PMA) and ionomycin (Sigma) for 5 h, followed by fixation and permeabilization using a commercial kit (e-Bioscience, San Diego, California, USA), according to the manufacturer’s instructions. Both interferon-γ (IFN-γ) and IL-17 antibodies were used for intracellular cytokine staining. Foxp3 expression was performed via the fixation and permeabilization procedure. The following monoclonal antibodies (mAbs) were used for extracellular staining: CD4, CD25, CD127 and GARP. The following mAbs were used for intracellular staining: IL-17, IFN-γ and Foxp3. For GARP transduction, cells were activated with CD3/CD28 beads (Invitrogen). All antibodies were purchased from BD Biosciences or Pharmigen (San Jose, CA, USA) and e-Bioscience (San Diego, CA, USA). Data acquired by flow cytometry were analyzed using FlowJo software (Tree Star).

### T-cell proliferation assays

CD4^+^CD25^−^ responder cells (5 × 10^4^) from healthy controls were stained with carboxyfluorescein succinimidyl ester (CFSE, Molecular Probes), and were cocultured with 5 × 10^4^ CD25^+^GARP^+^ Tregs from healthy control or COPD patients sorted by the Aria sorter in anti-CD3-coated (5 μg/mL OKT3 mAb; eBioscience) plates in the presence of soluble anti-CD28 (5 μg/mL; eBioscience) for 72 h. Proliferation of the cells was assessed on day four or day five by analyzing the dilution of CFSE in the cells with flow cytometry.

### Statistical analysis

Group data were expressed as the mean and S.E.M or as the median and interquartile range, where appropriate. For data distributed normally, one-way analysis of variance (ANOVA) with *post-hoc* multiple comparisons using the Tukey’s method was performed to determine significance of differences between groups. For data not distributed normally, comparisons between groups were made using one-way Kruskal–Wallis tests, and a p-value of < 0.05 was considered significant. If this test indicated significance, the Dunn’s multiple comparison test was used for *post-hoc* analysis for comparisons between two groups. Correlation was assessed by calculating Spearman's rank correlation coefficient. Here, a p-value of < 0.05 was considered significant. All significance levels were based on two-tailed tests. Statistical analyses were performed using Prism (GraphPad Prism 7.0).

## Results

### Demographic characteristics of study subjects

**Fig. 1:**
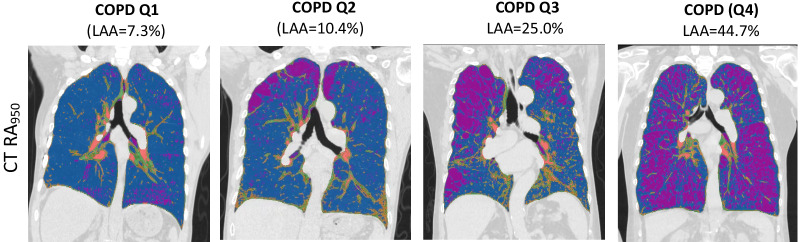
Central coronal chest CT emphysema quantification. The quantification of emphysema, based on the RA950, is demonstrated in the representative coronal CT scan from four individuals with varying severity of emphysema. From this classification, normal lung tissue is denoted blue, and emphysema is denoted purple. Patients with more advanced emphysema have more purple areas. RA950: relative area of the CT density histogram voxels < − 950 HU

Initially, 66 stable COPD patients were recruited but only those with full CT profile (n = 43) were included in the current analysis. The demographic characteristics of COPD patients enrolled in this study are shown in Table 1. All the patients with COPD were stratified by CT scan quantification of emphysema quartiles (Fig. 1 and Additional file 1: Fig. S1). Table 2 illustrates the main baseline clinical features of COPD patients included in the study, stratified by CT emphysema quartiles.

**Table 1:** Demographics and spirometry of all participants

	Never smokers	Smokers	COPD
*Subjects (n)*	14	12	43
*Demographics*			
Age (year)	65 ± 5	67 ± 6	66 ± 10
Male sex %	64	75	72
BMI (kg m^−2^)	28 ± 6	29 ± 6	25 ± 8
*Smoking status*			
Pack-years	0	37 ± 18	44 ± 23
Current smoker %	0	83	23
*Physiology*			
FEV1% predicted	94 ± 5	98 ± 8	50 ± 22
FEV1/FVC %	85 ± 4	82 ± 3	48 ± 16

Data are presented as median (IQR) for smoking status, mean ± SD for all others

FEV_1_, forced expiratory volume in 1 s; FVC, forced vital capacity

**Table 2:** Baseline characteristic of patients stratified by quartiles of CT emphysema severity

	Q1 LAA < 7.5%	Q2 LAA 7.5–15%	Q3 LAA 15–25%	Q4 LAA 25%	p-value (ANOVA)
*Subjects (n)*	9	11	13	10	
*Demographics*					
Age years	64 ± 9	65 ± 8	67 ± 7	66 ± 8	NS
Male sex %	68	70	78	76	< 0.001
BMI (kg m^−2^)	26 ± 7	27 ± 5	25 ± 6	23 ± 6	< 0.001
BMI < 21 kg m^−2^%	14	12	19	30	< 0.001
Smoking status					
Pack-years	41 ± 19	40 ± 21	43 ± 22	45 ± 19	< 0.01
Current smoker %	33	30	15	14	< 0.001
*Medications %*					
Inhaled steroids	15	25	56	52	< 0.001
Oral corticosteroids	0	0	0	0	NS
*Physiology*					
FEV_1_% pred	59 ± 13	52 ± 14	46 ± 14	44 ± 16	< 0.01
FEV_1_/FVC %	55 ± 9	51 ± 10	41 ± 9	43 ± 6	< 0.01
Oxygen saturation %	95.8 ± 2.9	96.2 ± 3.9	92.8 ± 3.4	91.4 ± 2.3	< 0.001
*Comorbidities (%)*					
Cardiovascular diseases	15	20	17	13	< 0.01
Osteoporosis	18	20	35	42	< 0.01
Diabetes	9	10	5	6	< 0.01
*Longitudinal outcomes*					
COPD hospitalizations PPPY*	0.4	0.8	1.6	3.4	< 0.001
COPD exacerbations PPPY*	1.2	2.6	3.4	3.8	< 0.001
FEV_1_ decline mL·year^−1^	28 ± 26	32 ± 30	35 ± 33	36 ± 32	< 0.01

LAA: low attenuation area; BMI: body mass index; FEV1: forced expiratory volume in the first second; FVC: forced vital capacity; PPPY: per patient per year

*Average number of events per year

In line with a previous report [23], patients with more severe emphysema were more likely to have lower BMI, worse oxygen saturation, FEV_1_, symptom and health status, and were more likely to be a heavy ex-smoker (Table 2). They were also more likely to report osteoporosis and less likely to report having diabetes or cardiovascular disease. Most subjects in all quartiles were receiving inhaled corticosteroid, whereas a minority of subjects (< 2%) were receiving oral corticosteroid (Table 1). Retrospective analysis showed that patients with more emphysema had a higher frequency of acute exacerbations, hospitalizations associated with accelerated lung function loss over the past two years.

### Selective expression of GARP on suppressive activated Treg Cells

**Fig. 2:**
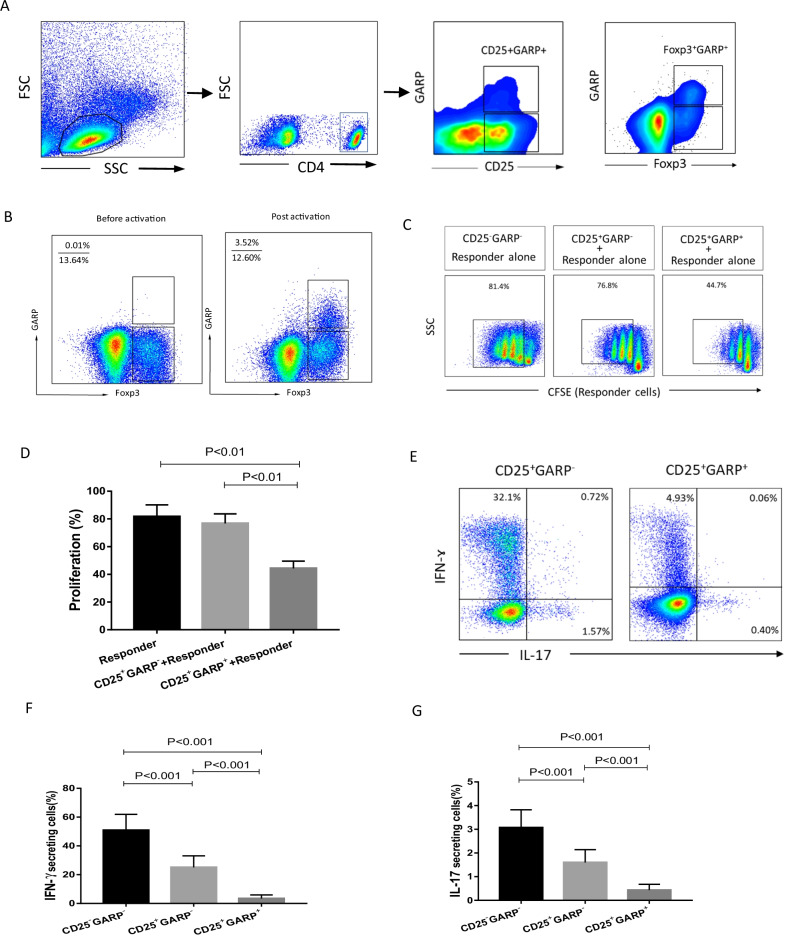
GARP specifically defines suppressive activated Tregs. **A** Gating strategy and representative flow cytometry plots of GARP expression are shown. GARP is selectively expressed in CD4^+^CD25^+^ and CD4^+^Fop3^+^ cells after TCR stimulation. CD25^+^GARP^+^ and CD25^+^GARP^−^ subpopulations can be sorted for in vitro suppressive function assay. **B** Induction of GARP on CD4^+^Fop3^+^ cells after TCR stimulation (anti-CD3 and anti-CD28 beads). **C** In vitro suppressive function assay of the CD25^+^GARP^+^ and CD25^+^GARP^−^ subsets. The evaluation of the suppressive function of each subset was performed by CFSE staining of the responder cells. Percentages of dividing responder cells per well were indicated. Data are representative of five separate experiments. **D** The percentages of CD25^+^GARP^−^ and CD25^+^GARP^+^ cells prohibiting proliferation in each group are shown in a summary graph. **E**Flow cytometry of the secretion of IFN-γ vs. IL-17 by GARP^−^ and GARP^+^ subsets after stimulation with PMA + ionomycin for 5 h. Summary graph showing the percentages of IFN-ɣ and IL-17 secreting cells in each subset, respectively (**F**, **G**). One-way ANOVA with post hoc pairwise multiple comparisons using the Tukey’s method. For **D**, **F** and **G**, data are from n = 5 independent experiments using T cells from different healthy donors (n = 5 subjects)

**Fig. 3:**
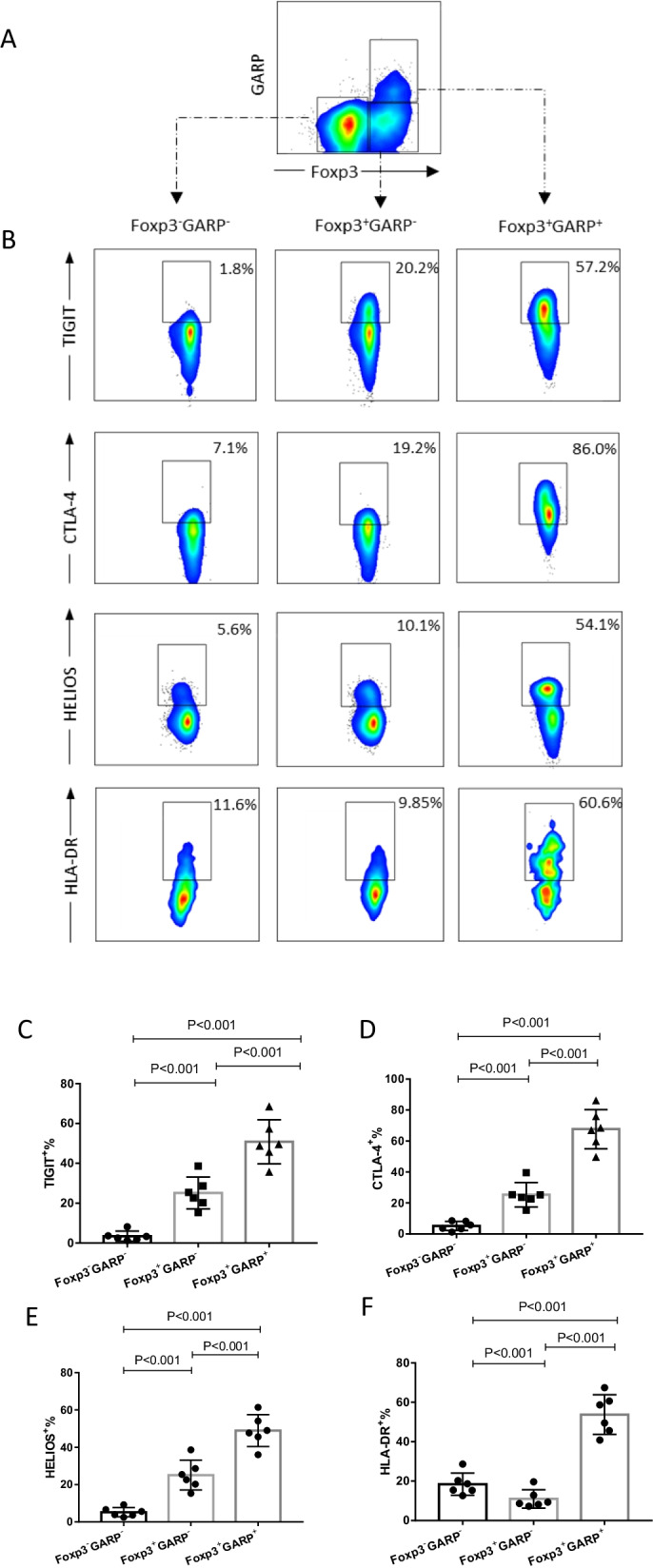
Functional markers relevant to nTregs are significantly expressed in Foxp3^+^GARP^+^ T cells. **A** Gating approach for CD4^+^ T cell subpopulations based on Foxp3 and GARP expression. **B** Representative flow cytometry plots showing the expression of classical Tregs markers in three CD4^+^ T cell subpopulations from healthy donors. **C** Statistical graphs depicting the expression of those markers in each subpopulation of CD4^+^ T cells

As described previously [12], GARP is specifically highly expressed in the CD25^+^ or Foxp3^+^ populations (Fig. 2A). Treg cells selectively express GARP only after TCR stimulation (Fig. 2B). To further investigate the potential to use GARP as a marker for activated Treg cells with suppressive capacity, CD25^+^GARP^+^ cells were sorted with cytometry after TCR stimulation to determine their functional activity. As Fig. 2C, D shows, CD25^+^GARP^+^ cells can more strongly inhibit the proliferation of responder cells compared to CD25^+^GARP^−^ cells. Remarkably, a much higher amount of pro-inflammatory cytokines, including IL-17 and IFN-γ were detected in CD25^+^GARP^−^ cells in response to PMA stimulation (Fig. 2E–G), which identified this subpopulation as the previously reported pro-inflammatory cytokines secreting Tregs [24]. Compared with CD25^+^GARP^−^ cells, the CD25^+^GARP^+^ population showed a higher expression of TIGIT, CTLA-4, HELIOS, and HLA-DR (Fig. 3A–F) suggesting that these cells are a proliferating, activated fraction of Tregs. Taken together, GARP not only defines activated regulatory T cells with highest inhibitory capacity but also discriminate CD25^+^Foxp3^+^ T cells that secrete pro-inflammatory cytokines.

### TGF-β can induce Foxp3 expression but not GARP expression

**Fig. 4:**
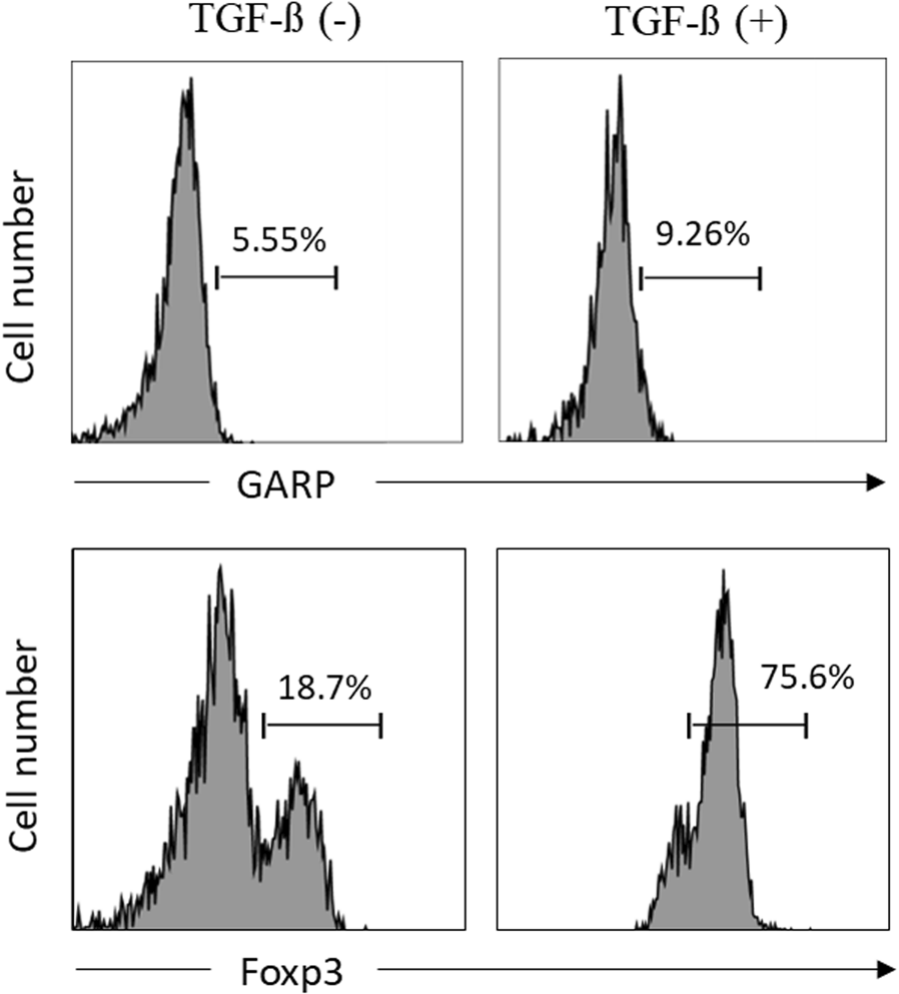
TGF-β treatment leads to different induction of Foxp3 and GARP in T_N_ cells. T_N_ cells were activated via TCR (CD3CD28 beads) with or without 20 ng/mL TGF-β. Foxp3 and GARP expression were measured after two days of activation

It has been shown that TGF-β induces Foxp3 expression in both murine and human T cells [8, 9], but unlike murine cells, TGF-induced human CD25^+^Foxp3^+^ cells do not confer a regulatory phenotype. Consistent with previous studies [12], we further determined that TGF-β induce Foxp3 expression but not GARP expression (Fig. 4), suggesting the transcription factor Foxp3 is not essential for the induction of GARP and GARP can truly distinguish activated nTreg cells from induced Treg cells and activated T cells that have no immunosuppressive function.

### Decreased frequencies of circulating Foxp3^+^GARP^+^ Tregs associated with multi-organ loss of tissue phonotype

**Fig. 5:**
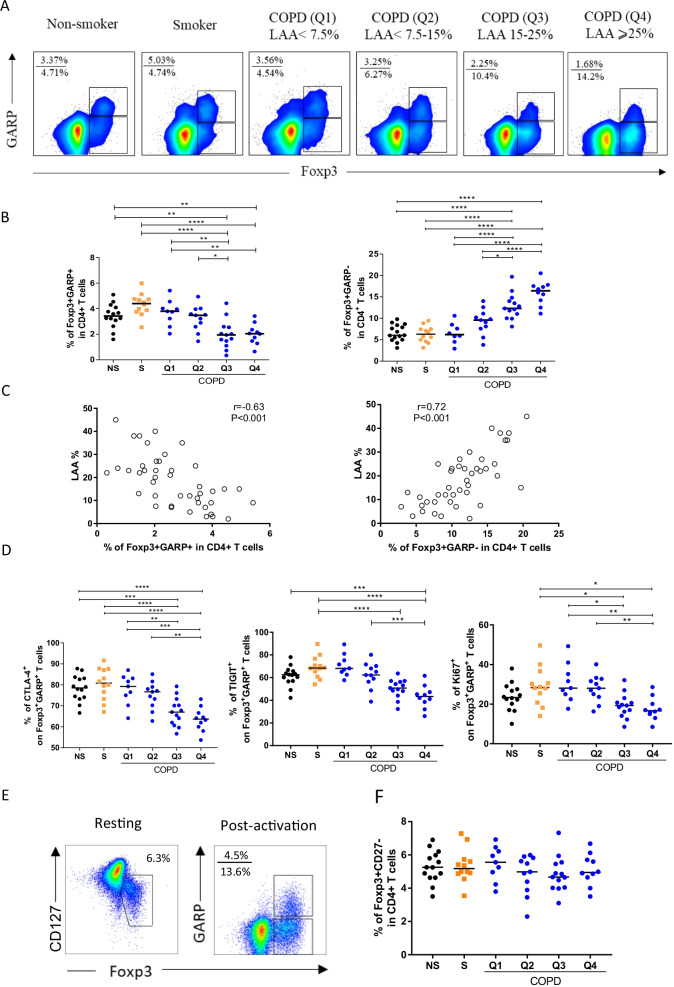
GARP expression on CD4^+^Foxp3^+^ T cell are progressively decreased in different severity of emphysema. **A** Representative flow cytometry plots of GARP expression are shown. **B** The proportions of Foxp3^+^GARP^+^ and Foxp3^+^GARP^−^ subsets among CD4^+^ T cells in all subjects. **C** Negative statistical correlation between frequency of circulating FoxP3^+^GARP^+^ and LAA% (**C**, Left). Positive statistical correlation between frequency of circulating FoxP3^+^GARP^−^ cells and LAA% (**C**, Right). r = − 0.63, p < 0.001 and r = 0.72, p < 0.001 by Pearson’s correlation. **D** Expression of Treg cell function-specific markers (CTLA-4 and TIGIT) and activation markers (Ki-67) in peripheral FoxP3^+^GARP^+^ cells. One-way ANOVA with post-hoc pairwise multiple comparisons using Tukey’s method. *p < 0.05; **p < 0.01; ***p < 0.001; ****p < 0.0001. **E** Representative staining of CD4^+^ T cells for GARP, CD127, and FOXP3 expression. CD4^+^ T cells were stained for CD127 and FOXP3 expression as resting T cells and for GARP and FOXP3 expression following 1 day of TCR activation. **F** Analysis of Foxp3^+^CD127^−^ T cells from all subjects

We analyzed circulating Foxp3^+^GARP^+^ cells in non-smoker, smokers, and COPD subjects at different stages of disease. Cytometric assays revealed that healthy smokers had a higher frequency of Foxp3^+^GARP^+^ Tregs compared with non-smokers and COPD subjects (Fig. 5A, B). In COPD patients, the frequency of Foxp3^+^GARP^+^ Treg subsets progressively decreased from Q1 to Q4, whereas the frequency of Foxp3^+^GARP^−^ cells increased from Q1 to Q4 (Fig. 5A, B). Not surprisingly, the frequency of Foxp3^+^GARP^+^ Tregs were inversely correlated with the LAA (r = − 0.63, P < 0.001) whereas the frequency of Foxp3^+^GARP^−^ cells correlated with LAA of COPD patients (r = − 0.72, P < 0.001) (Fig. 5C). In addition to the emphysema quartiles, we also analyzed circulating Foxp3^+^GARP^+^ Tregs in COPD patients staged using the Global Initiative on Obstructive Lung Disease (GOLD) criteria and compared them with both non-smoker and smoker controls. No significant reduction in the frequency of Foxp3^+^GARP^+^ subsets was found in COPD patients at different GOLD grades. Furthermore, there was no statistically significant difference in Foxp3^+^GARP^−^ cells of COPD patients as they progressed from GOLD II to IV (Additional file 1: Fig. S2).

Next, we measured markers associated with suppressive (CTLA-4 and TIGIT) and proliferative function (Ki-67) on Tregs. In line with the expression trend of GARP, the proportion of CTLA-4, TIGIT and progressively decreased from Q1 to Q4 in COPD patients. However, the patients with COPD at Q1 and Q2 expressed higher levels of Ki-67 than the patients at Q3 and Q4 (Fig. 5D). Taken together, the results indicate that the expression levels of functional markers on the surface of Treg cells are normal or mildly increased in the early stages of the disease, and these markers progressively decrease as the disease progresses.

### Progressive changes of Treg cell function during COPD development

**Fig. 6:**
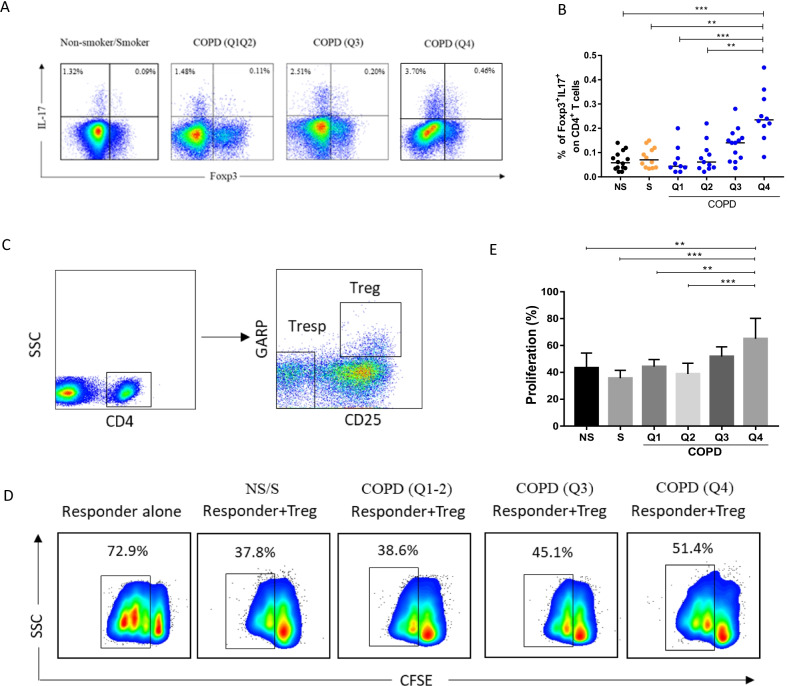
Impaired suppressive function and enhanced IL-17 production of Tregs from COPD patients. **A** Representative intracellular staining of Foxp3 vs. IL-17 in non-smoker (n = 6), smoker (n = 6), and COPD patients (n = 6) at a different quartile after 5 h of stimulation with PMA/ionomycin and GolgiStop. **B** The proportions of Foxp3^+^IL-17^+^ subsets among CD4^+^ T cells in all subjects. One-way Kruskal–Wallis test with *post-hoc* multiple comparisons using the Dunn’s method. **p < 0.01; ***p < 0.001; **C** CD25^+^GARP^+^ Tregs and CD25^−^GARP^−^ responder T cells (Tresp) were isolated by FACS. For suppression and proliferation assays, CFSE-labeled Tresp cells were co-cultured with Treg cells with plate-bound anti-CD3 and soluble anti-CD28 for 96 h, alone or in 1:4 ratios. Tresp cells were isolated from non-smoker and Tregs were isolated from non-smoker, smoker and COPD patients in different quartiles. **D** Suppressive function of Tregs decreased with the CT scan quantification quartile. The flow cytometry plot shows dilution of CFSE labeled responder cells. The percentage of dividing cells is shown. **E** Suppressive function assay revealed that CD25^+^GARP^+^ Treg cells from COPD patients have a reduced suppressive capacity and associated with the severity of emphysema. Data are from n = 8 independent experiments (at least n = 3 subjects in 2 technical replicates). One-way ANOVA with post hoc pairwise multiple comparisons using Tukey’s test **p < 0.01; ***p < 0.001

The altered GARP expression prompted us to perform in vitro experiments to further evaluate the immune function of nTregs in COPD patients. Consistent with the immune phenotype results, the frequency of Foxp3^+^ Il-17-secreting cells were increased in COPD patients and associated with the severity of emphysema (Fig. 6A, B). However, the immuno-suppressive capacity of Foxp3^+^ Tregs tends to decrease progressively with increasing emphysema severity (Fig. 6C–E). Together, these finding suggest that the compromised immunomodulatory capacity and enhanced pro-inflammatory capacity are involved in lung tissue destruction in COPD.

### Is GARP Expression a More Accurate Marker to Detect Tregs During Chronic Inflammation in COPD?

Reliably discriminating Tregs from activated T cells is challenging, because Tregs markers are potentially modified during T-cell activation or differentiation, especially under chronic inflammation [11]. That prompted us to look into whether GARP could be a better marker for identifying bona fide Tregs in the chronic immune activation of COPD. Using the classic definition, the percentage of Tregs in peripheral blood of different populations was examined. Treg identification using CD4^+^ Foxp3^+^CD127^−^ (Fig. 5E, F) revealed no differences in the frequency of circulating Tregs in patients with COPD compared to smokers and never-smokers, confirming prior research [25, 26]. Combined with in vitro functional assays, this striking discordance between the frequency of FOXP3^+^CD127^−^ T cells and FOXP3^+^GARP^+^ T cells suggest that GARP expression can better identify Tregs during systemic inflammation in COPD.

### The similar pattern of GARP variation in BAL

**Fig. 7:**
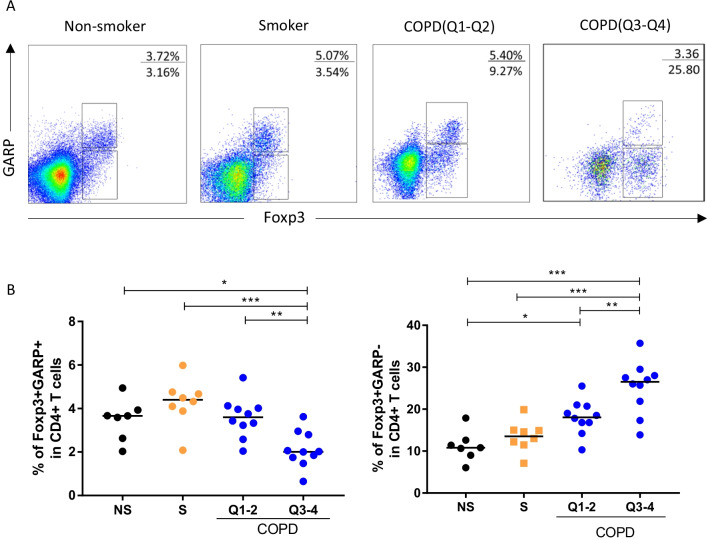
Decreased Foxp3^+^GARP^+^ Tregs in BAL of COPD patients. **A** Representative intracellular staining of Foxp3 vs. GARP in BAL fluid from non-smoker (n = 7), smoker (n = 6) and COPD patients (n = 15) at a different quartile. **B** The proportions of Foxp3^+^GARP^+^ Tregs and Fop3^+^GARP^−^ T cell among CD4^+^ T cells in all subjects. One-way ANOVA with post-hoc pairwise multiple comparisons using the Tukey’s test *p < 0.05; **p < 0.01; ***p < 0.001

To clarify the GARP expression of pulmonary Tregs, BAL samples were obtained from seven never-smokers, eight smokers and twenty COPD patients having clinical investigational bronchoscopy. Interestingly, we found strong GARP expressions of Tregs in BAL without activation of CD3CD28 beads. Foxp3^+^GARP^+^ Tregs in BAL shows a similar pattern of variation in peripheral blood, that is, frequencies of Foxp3^+^GARP^+^ Tregs decreased whereas Foxp3^+^GARP^−^ T cells increased in COPD patients and associated with the severity of multi-organ loss of tissue phonotype (Fig. 7A, B).

## Discussion

As the most common chronic inflammatory respiratory disease, COPD is characterized by airflow limitation that is not fully reversible. Cellular and molecular mechanisms involved in this disease remain ill-defined. nTregs play a critical role in controlling immune responses and maintaining immune homeostasis. It has been shown, alterations in the frequencies and/or function of nTregs have great influence over the development and progression of disease. However, lack of an exclusive marker for defining bona fide nTregs is a major obstacle for understanding their clinical relevance in COPD. Foxp3 and other surface markers of Tregs, such as CD25, CD62L, GITR, CTLA-4 and CD127 also upregulate or downregulate upon T cell activation [5, 27]. GARP has been shown to be a highly specific molecular marker for activated nTregs but absent on activated or inactivated effector T cells. Building on this novel definition of nTregs, we revealed that peripheral Foxp3^+^GARP^+^ Tregs are progressively reduced whereas Foxp3^+^GARP^−^ population progressively increased with increasing quartiles of CT emphysema severity in COPD. We also noted that patients with greater emphysema experience greater loss of pulmonary and extrapulmonary tissue. Those findings are in keeping with one previous research that report a multi-organ loss of tissue phenotype. Our study linked the immune phenotype with the clinical phenotype of COPD.

TGF-β is a potent immunosuppressive cytokine that regulate immune response. GARP, the membrane binding receptor of latent-TGF-β [28], plays a pivotal role in promoting TGF-β activation by enabling the interplay of TGF-β with integrins αvβ6 or αvβ8 [29], which is thought to be a primer step for releasing bioactive TGF-β. Tregs are a major source of TGF-β, and GARP expression on Tregs is essential for keeping their immunosuppressive functions [18]. In addition to Tregs, GARP is also present on the surface of activated platelets, B cells, hepatic stellate cells and mesenchymal stem cells [30]. In addition, GARP can be secreted in the plasma as soluble GARP [31]. In contrast to FoxP3, GARP is expressed by a wider variety of cells in an immunosuppressive milieu. Numerous studies suggest that GARP can be used as a biomarker for disease prediction and therapeutic follow-up in a variety of cancers and blocking GARP-TGF-β interaction could be a potential therapeutic target in cancer. A phase I clinical trial (NCT03821935) for an antibody against GARP is underway in clinics.

Immune imbalance caused by altered Treg cell numbers or function due to long-term smoking is important for a profound understanding of COPD development and progression. However, assessing the holistic deficiencies of Tregs in COPD has been a big challenge and previous research revealed inconsistent results due to lack of an exclusive marker for Treg and heterogeneity of disease. In contrast to cancer, research on Tregs in COPD is still in its infancy. Using GARP, the most specific marker of Tregs to date, we aim to bring more accurate evaluation of immunomodulatory function to COPD. In our study, as the severity of emphysema increased, the frequency of Foxp3^+^GARP^+^ gradually decreased, while Foxp3^+^GARP^−^ continuously increased, suggesting that compromised immunomodulatory capacity and enhanced pro-inflammatory capacity are involved in the pathogenesis of emphysema. This dynamic change of GARP gives us a picture of immune exhaustion due to long term smoking exposure and a progressively increasing systemic inflammatory response, which is associated with multi-organ tissue loss in the COPD patients.

It is of interest to note, IL-17-secreting Tregs, contained within the Foxp3^+^GARP^−^ population, increased with the severity of emphysema in our study. However, the final differentiation of these subpopulation remains unclear, with the possibility of switching to the Th17 cells or possessing dual Treg/Th17 characteristics. Given Th17 plays an important role in COPD [1, 32], we speculate that this cell subpopulation is in an intermediate state of differentiation from Treg cells to Th17 cells and may be related to the imbalance between Th17 cells and Tregs in COPD. Further study is needed to clarify the role of this subpopulation in the mechanism of COPD.

COPD is being increasingly regarded as a marked heterogeneous disorder, with distinct pathobiology. Exploring the immune mechanisms behind different clinical phenotypes offers a very interesting research strategy to understand the complexity of COPD. The original descriptions of the different clinical phenotypes of COPD can be dated back to the 1950s, with the emphysema-dominant phenotype often referred to as "pink puffer" and the bronchitis-dominant phenotype referred to as "blue bloater". More recently, a novel term MOLT was coined based on refinement of the original “pink puffer” phenotype. Patients with MOLT phenotype have more emphysema, undergo an excessive loss of tissue in multiple organs including lungs, bones, adipose tissue, and skeletal muscle, and are more likely to have acute exacerbations and higher mortality over time [23, 33]. These suggest that the immunological mechanisms behind the different clinical phenotypes or stages of COPD may differ, and it is important to clarify these specific mechanisms for future individualized immunotherapy.

It is assumed that the possible mechanism underlying the multiple organ loss is the systemic autoimmune response triggered by cigarette smoking [34]. Tregs play a pivotal role in the development of the inflammation. Our results suggested compromised activated nTregs allow the persistent pulmonary and systemic inflammation. However, the underlying molecular and cellular pathways remain unclear. Based on the foregoing [35], it can be speculated that the microenvironmental stimuli generated by cytokine release and immune metabolism during COPD progression led to a dynamic decrease in GARP.

The present study had several limitations. First, only peripheral blood and BAL samples were studied. It would be interesting to further characterize Tregs on lung tissues, because the role of Treg cells in regulation of the immune response may vary in different lung regions^36^ and this requires further clarification. Secondly, the results of the current cross-sectional study only indirectly suggest variation of GARP involved in the development of emphysema; Prospective and in vitro studies are still needed to clarify the role of GARP in the mechanism of emphysema.

In summary, this study demonstrates that decreased highly suppressive nTreg, defined by the GARP^+^ subpopulation, and accumulated pro-inflammatory cytokine-producing Tregs, defined by the GARP^−^ subpopulation, reflect more advanced disease in the MOLT phenotype of COPD. GARP may represent an intriguing biomarker for disease evaluation and has the potential to act as a target to regulate the function of Tregs in COPD. Our data link the immune phenotype with clinical phenotype which may contribute to a better understanding of the underlying pathogenesis of inflammation in COPD.

## Supplementary Information

**Additional file 1: Figure S1.** COPD patients were stratified by quartiles of computed tomography emphysema severity. Data are expressed as mean ± SEM. **P < 0.01; ***P < 0.001; ****P < 0.0001 by two-tailed Mann–Whitney test. **Figure S2.** GARP expression on CD4^+^Foxp3^+^ T cell in COPD patients with different GOLD stages. Cumulative data of CD4^+^FoxP3^+^GARP^+^ (A) and CD4^+^FoxP3^+^GARP^−^ (B) cells in PBMCs from non-smoker, Smoker, and COPD subjects at various GOLD stages. One-way ANOVA with post-hoc pairwise multiple comparisons using Tukey’s method. *p < 0.05; **p < 0.01; ***p < 0.001; ****p < 0.0001.

## Data Availability

Data and materials are available upon request by contacting the correspondence author Jia Hou (houj@nxmu.edu.cn).

## Abbreviations

GARP: Glycoprotein A dominant repeat
COPD: Chronic obstructive pulmonary disease
BMI: Body mass index
TCR: T cell receptor
CFSE: Carboxyfluorescein succinimidyl ester
nTregs: Naturally occurring regulatory T cells
LAA: Low attenuation area
