# Supplementary figures and images for: Reduced frequencies of Foxp3+GARP+ regulatory T cells in COPD patients are associated with multi-organ loss of tissue phenotype
Source: Respir Res. 2022 Jul 2;23:176. doi: 10.1186/s12931-022-02099-2 (PMC9250745; doi:10.1186/s12931-022-02099-2)

## Slide 1
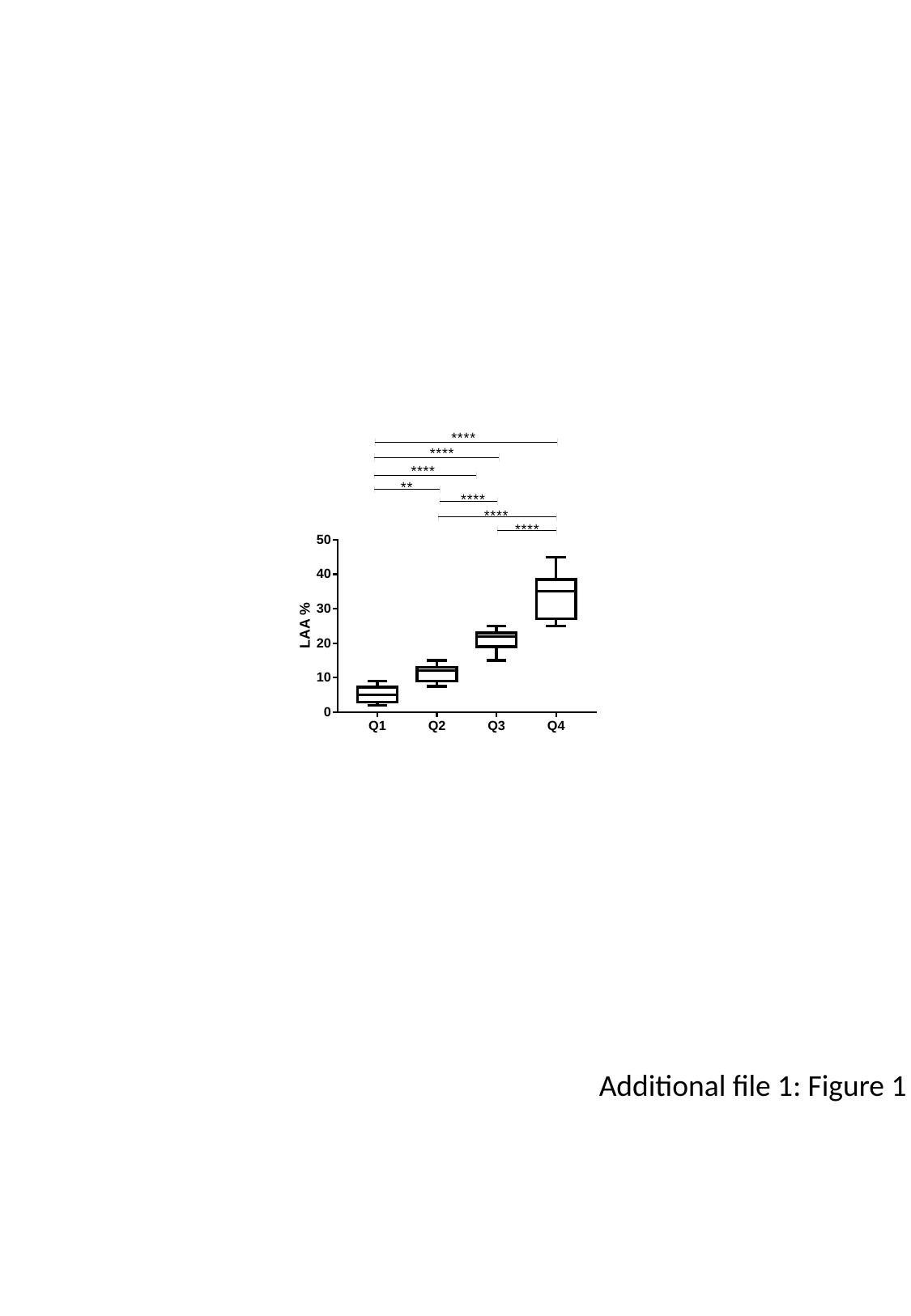

Additional file 1: Figure 1

## Slide 2
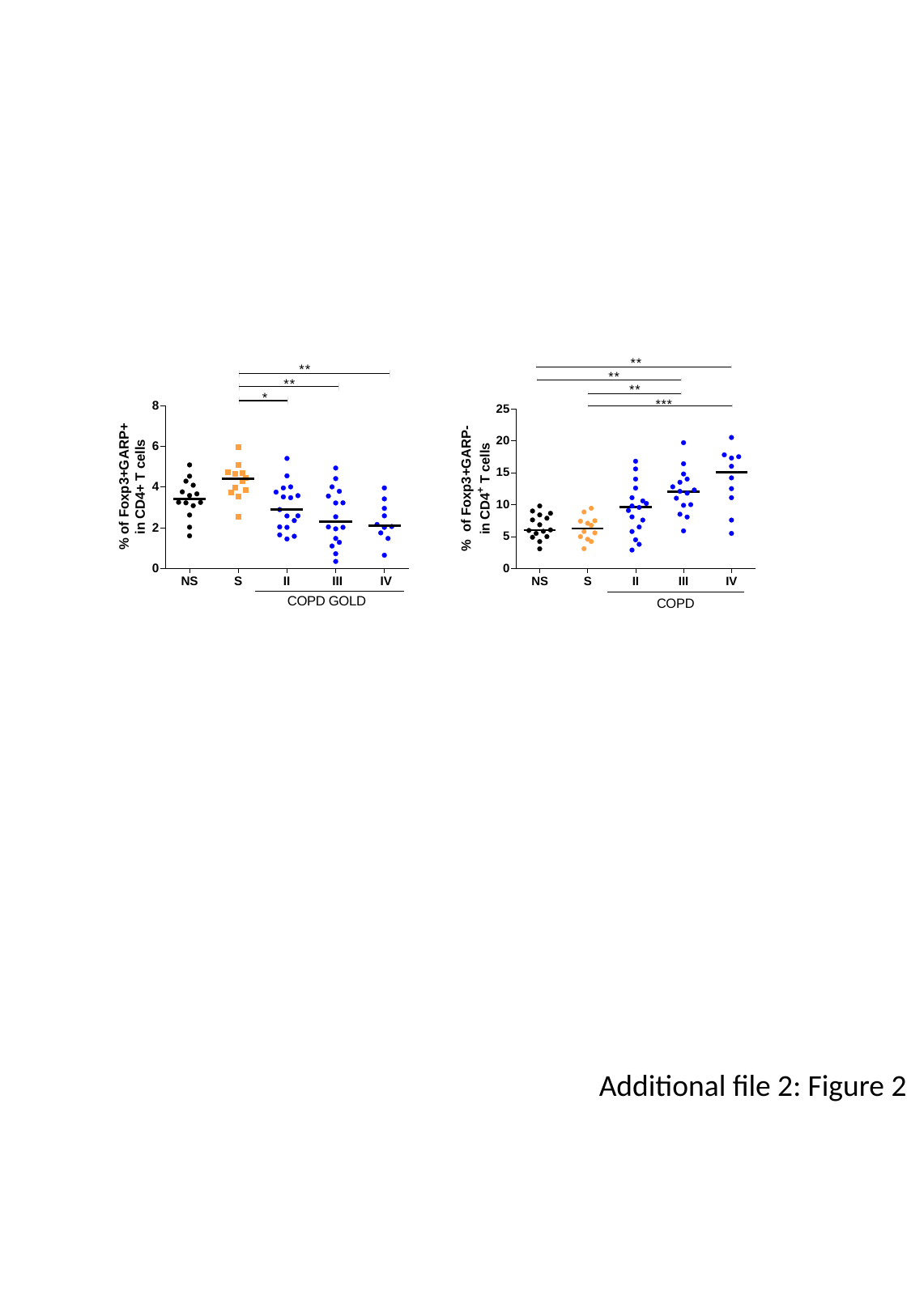

Additional file 2: Figure 2

Supplement: Supplementary file 1 — Additional file 1: Figure S1. COPD patients were stratified by quartiles of computed tomography emphysema severity. Data are expressed as mean ± SEM. **P < 0.01; ***P < 0.001; ****P < 0.0001 by two-tailed Mann–Whitney test. Figure S2. GARP expression on CD4+Foxp3+ T cell in COPD patients with different GOLD stages. Cumulative data of CD4+FoxP3+GARP+ (A) and CD4+FoxP3+GARP− (B) cells in PBMCs from non-smoker, Smoker, and COPD subjects at various GOLD stages. One-way ANOVA with post-hoc pairwise multiple comparisons using Tukey’s method. *p < 0.05; **p < 0.01; ***p < 0.001; ****p < 0.0001. [file 12931_2022_2099_MOESM1_ESM.pptx]
